# Supplementary material for: The impact of municipal solid waste sorting policy on air pollution: Evidence from Shanghai, China
Source: PLoS One. 2022 Nov 2;17(11):e0277035. doi: 10.1371/journal.pone.0277035 (PMC9629625; doi:10.1371/journal.pone.0277035)
Supplement: Attachment — Submitted filename: Response to Editor.docx [file pone.0277035.s002.docx]

Response to Editor

Dear Associate Professor Ghaffar Ali

Thank you very much for giving us the chance to revise and resubmit the manuscript. We truly appreciate the valuable comments and suggestions that have helped substantially improve the quality of the paper.

Based on the submission guidelines and map copyright guidelines you sent to us, we carefully changed the style form of this manuscript to meet the journal requirements: 1. Moved the content of footnotes into the main text and deleted footnotes; 2. Changed the Reference list into “Vancouver” style; 3. Made the line spacing, abbreviations as well as equations meet the style requirments, etc. We also rebuilt Fig 3 by using the map and data from OpenStreetMap site that are compatible with CC BY licensing and added the map statement in Acknowledge. The new Fig 3 is as follows:

*Note: Base map and data from OpenStreetMap and OpenStreetMap Foundation,* *original copyright:* *<https://www.openstreetmap.org/copyright>. The stars mark the locations of air monitoring stations,* *and the dots mark the locations of domestic waste incineration plants in Shanghai.*

We have uploaded figure files to the Preflight Analysis and Conversion Engine (PACE) digital diagnostic tool, https://pacev2.apexcovantage.com/.

Following the Reviewer’s comments, we have made the following major revisions to the paper. Note that we would not like to present some minor revisions to minimize the length of the letter.

1. *“This paper looks like a report rather a scientific paper. Authors should have consulted authors' guidelines carefully before submission.“*

We have carefully changed the style form of this manuscript to meet the journal requirements. We have reorganized the structure of this manuscript and rewritten the introduction section by directly stating our research question and addressing how our work contributes to the literature.

Specifically, the revised chapter structure is:

1.Introduction

2.Materials and Methods

2.1 Policy Background and Research Hypotheses

2.2 Data Source and Description

2.3 Empirical Strategy

3.Results

3.1 Baseline Findings

3.2 Parallel Trend Test

3.3 Robustness Tests

3.4 Spillover Effect

4.Discussion

5. Conclusions

Acknowledgments

References

*2. “Another issue lies with novelty, no clear hypothesis or research questions to test.”*

This comment is very enlightening. We have improved the manuscript in two ways. First, in the **Introduction** section, we directly state our research question after providing background. Then, we address how our work contributes to the literature. Second, we reorganized the structure of this manuscript, adding a subsection: **2.1 Policy Background and Research Hypotheses** to clearly propose the three research hypotheses in this study.

Specifically, the research questions are:

Therefore, in this study, we focus on the questions of whether the MSW sorting policy in Shanghai significantly improves urban air quality and how large the influence will be.

The three research hypotheses are:

**H1:** The MSW sorting policy in Shanghai will reduce the air pollutants generated by waste incineration plants and significantly improve the air quality in Shanghai.

**H2:** The degree of influence on air quality will be affected by the distances to waste incineration plants. The closer to waste incineration plants, the larger the influence will be.

**H3:** There will be a positive spillover effect on the air quality in adjacent cities.

*3.“Many subsections are unorthodox. I do not know why need to add such headings rather follow a standard pattern.“*

We have reorganized the structure of this manuscript and made each heading the same as the requirements of the Manuscript Organization in journal guidelines.

Specifically, the main modifications include:

Changed the “2. Policy Background” into a subsection in 2.Materials and Methods “2.1 Policy Background and Research Hypotheses”;

Changed the “4.2 Common Trend Test” into “Parallel Trend Test”;

Changed the “6. Conclusion and Policy Implications” into “5. Conclusions”

4.“*Positive externalities are given, how about negative?“*

We have added a new paragraph in the **Discussion** section to address the immature aspects of the MSW sorting policy in Shanghai, which has caused inconvenience to residents.

Finally, there are still many immature aspects of the MSW sorting policy in Shanghai, which has caused inconvenience to residents. Wu et al. (2021) [34] used a text mining technique to uncover attitudes from the Chinese public toward MSW sorting policies. They find that although a large proportion of the Chinese public has a positive attitude toward the MSW sorting policy, the proportion of people with negative emotions reached nearly half. Most negative emotions were toward fines, MSW sorting rules, fees, timing of throwing waste, and irregular recycling procedures. Our parallel trend test also shows that the impact of the MSW sorting policy seems to become weaker in the long term. Therefore, to maintain the sustainability of MSW sorting behaviors, the government needs to further improve efficiency and reduce residents' time costs.

5.“*Revise Figure 3 for accuracy and better map/location/resolution, etc. In addition, few other issues such as report style write up and settings.“*

We have rebuilt Fig 3 as above. We also avoided the writing style of the report by removing some unnecessary statistics listed in the **Introduction** section, removing the policy implications in the **Conclusion** section and using an editing tool to improve the write up of this manuscript.
